# Are Personal Health Records Safe? A Review of Free Web-Accessible Personal Health Record Privacy Policies

**DOI:** 10.2196/jmir.1904

**Published:** 2012-08-23

**Authors:** Inmaculada Carrión Señor, José Luis Fernández-Alemán, Ambrosio Toval

**Affiliations:** ^1^Research Group of Software EngineeringDepartment of Informatics and Systems, Faculty of Computer Science, Regional Campus of International Excellence “Campus Mare Nostrum”University of MurciaMurciaSpain

**Keywords:** Personal health records, privacy, computer security, standards, HIPAA, Health Insurance Portability and Accountability Act

## Abstract

**Background:**

Several obstacles prevent the adoption and use of personal health record (PHR) systems, including users’ concerns regarding the privacy and security of their personal health information.

**Objective:**

To analyze the privacy and security characteristics of PHR privacy policies. It is hoped that identification of the strengths and weaknesses of the PHR systems will be useful for PHR users, health care professionals, decision makers, and designers.

**Methods:**

We conducted a systematic review using the principal databases related to health and computer science to discover the Web-based and free PHR systems mentioned in published articles. The privacy policy of each PHR system selected was reviewed to extract its main privacy and security characteristics.

**Results:**

The search of databases and the myPHR website provided a total of 52 PHR systems, of which 24 met our inclusion criteria. Of these, 17 (71%) allowed users to manage their data and to control access to their health care information. Only 9 (38%) PHR systems permitted users to check who had accessed their data. The majority of PHR systems used information related to the users’ accesses to monitor and analyze system use, 12 (50%) of them aggregated user information to publish trends, and 20 (83%) used diverse types of security measures. Finally, 15 (63%) PHR systems were based on regulations or principles such as the US Health Insurance Portability and Accountability Act (HIPAA) and the Health on the Net Foundation Code of Conduct (HONcode).

**Conclusions:**

Most privacy policies of PHR systems do not provide an in-depth description of the security measures that they use. Moreover, compliance with standards and regulations in PHR systems is still low.

## Introduction

In many countries, it is tedious for patients to obtain copies of their official health records from health care providers, which makes it difficult for patients to seek second opinions or control their own information [1]. Moreover, records that patients create themselves tend not to be included in the official patient record. A personal health record (PHR) system can be maintained by patients and their families, can be shared with clinicians, and can support the maintenance of accurate and complete health records [1].

A PHR is “an electronic record of an individual’s health information by which the individual controls access to the information and may have the ability to manage, track, and participate in his or her own health care” [2]. A PHR should include all relevant information about the user’s life, including the following items: problem list, procedures, major illnesses, allergy data, home-monitored data, family history, social history and lifestyle, immunizations, medications, laboratory tests, and genetic information [3-5].

A PHR can take multiple forms: an independent software application running on a single computer; a Web service belonging to a single organization; a general Web service as a platform with which to collect different types of health information; or a USB-based PHR [6,7]. Maintaining data privacy is difficult in both PHRs and electronic health records (EHRs) [1], to the extent that, for instance, administrative staff could access information without the patient’s explicit consent [8]. Consumer concerns regarding PHR systems were found to be focused on two major areas: privacy and security [9]. A total of 91% of surveyed Americans stated that they were very worried about the privacy and security of their health information [7,9]. The aim of this review is to answer the following research question: What security and privacy features do PHR systems have? We carried out an in-depth analysis of many significant issues related to the security and privacy features of PHR privacy policies. The data collected were contrasted by analyzing the privacy aspects of 50% of PHR systems.

## Methods

The methods used to carry out the review were guided by a protocol. Iterative decisions concerning data collection, fields for extraction, analysis, and other relevant aspects of the survey were discussed in meetings that were attended and documented by the authors.

### Review and Protocol

This review followed the quality reporting guidelines set out in the Preferred Reporting Items for Systematic Reviews and Meta-analyses (PRISMA) statement [10].

### Eligibility Criteria

We used the following inclusion criteria (IC): (1) IC1: free PHR systems, (2) IC2: PHR systems with a Web-based format, and (3) IC3: patient-centered PHR systems with a privacy policy.

Based on the International Organization for Standardization (ISO) standard ISO/TR 12773 (*Business Requirements for Health Summary Records*), a PHR is defined as an electronic, universally available, lifelong resource of health information maintained by individuals, as opposed to an EHR, which is a repository of health information gathered across the longitudinal electronic record of the patient. This information is generated by one or more encounters in any care delivery setting [11]. Among the current variety of PHR support technologies, we focused our study on Web-based, free PHR systems. Free PHR systems can be used by anyone and are easiest to access (IC1). Web-based PHRs have certain benefits with regard to the use of the Internet (IC2) [11]. Moreover, the US Institute of Medicine recommended that “access to care should be provided over the Internet, by telephone, and by other means in addition to in-person visits” [12], while the 2003 Health Information National Trends Survey indicated that consumers use the Internet to access health information more often than they obtain this information from their health care professionals [13]. In addition, the number of users who use the Internet to access and manage their PHR is increasing [14-18]. Finally, according to the ISO, the owner of the record in a PHR system can be the health care organization, provider, or patient [11]. We also stipulated that the PHR systems included in the review should be patient-centered applications—that is, according to the definition of a PHR in the Health Insurance Portability and Accountability Act (HIPAA) [2], the information should be totally or partially managed by the patient (IC3). We analyzed this type of PHR system because they are more flexible and useful than non-patient-centered PHR systems, although they can have more privacy and security problems.

### Information Sources

We used two information sources: the myPHR website and scientific databases. The myPHR website was created by the American Health Information Management Association and contains information related to the use and creation of PHRs. To the best of our knowledge, this website provides the most comprehensive list of PHR systems that a user can find and has also been used to select PHR systems in multisource sampling [19]. Although our primary source was myPHR, we identified other PHR systems by reading articles extracted from the Medline, ACM Digital Library, IEEE Xplore Digital Library, and ScienceDirect databases, which we searched between February and April 2011. A systematic review was then used to review the articles indexed in these databases.

### PHR System Selection

The PHR system selection process was organized in the following six phases:

1. The search for PHR systems from the myPHR website.

2. The search for PHR systems from scientific databases. This phase was performed by means of a systematic review with the following search string: (“PHR providers” OR “Microsoft HealthVault” OR “Google Health”), which we adapted to database search engines. We next explored the articles identified in order to find the names of Web-based PHR systems.

3. Exploration of the PHR systems found, and a selection based on eligibility criteria IC1 and IC2.

4. Exploration of the PHR websites identified in order to find each one’s privacy policy and find out whether the PHR systems were patient-centered applications (eligibility criteria IC3).

5. A complete reading of each of the PHR privacy policies selected in the previous phase to extract their principal privacy and security characteristics.

The activities defined above were carried out by two authors independently. Disagreements were resolved by a third member of the team. The PHR system selection was developed in an iterative process of individual assessments until the interrater reliability was acceptable (0.9). In statistics, interrater reliability is the degree of agreement among raters, which gives a score with the level of consensus of the judges. We use the Cohen kappa coefficient for measuring this agreement. The Cohen kappa [20] coefficient is a statistical measure of interrater reliability for qualitative (categorical) items. A value of 0.9 indicates almost perfect agreement between the two privacy policy assessments performed by two authors.

### Data Collection Process

We collected data by using a data extraction form. The PHR system privacy policies were used to extract the methods employed to maintain the privacy and security of the users’ data. The privacy policy had to satisfy the security safeguards that are appropriate to the sensitivity of the information. They are used to protect personal information, according to Yee and Korba [21]. Note that Beldad et al [22] state that the omission of an assurance of security in a privacy statement may cause users to think that their personal data are susceptible to potential abuse, and this could discourage them from supplying the personal data needed to complete an online transaction. According to Earp et al [23], it is for this reason that online privacy statements often emphasize the application of security measures and the methods used for the collection of data.

### Data Items

In this study, we analyzed security and privacy of PHR systems in reference to the ISO 13606 standard [24]. Security was analyzed in terms of availability, confidentiality, integrity, and accountability. According to the ISO 13606 standard (*Electronic Health Record Communication Part 4: Security*), *availability *refers to the “property of being accessible and useable upon demand by an authorized entity.” This standard defines *confidentiality *as the “process that ensures that information is accessible only to those authorized to have access to it.” *Integrity *refers to the duty to ensure that information is accurate and not modified in an unauthorized fashion. *Accountability *refers to a person’s right to criticize or ask why something has occurred. The other topic analyzed in this study, privacy, has been defined as “the claim of individuals, groups, or institutions to determine for themselves when, how, and to what extent information about them is communicated to others” [25]. The characteristics analyzed in the privacy policies allowed us to analyze how privacy, integrity, and confidentiality are maintained.

We designed a template for the data to be extracted from each PHR system. In total, 39 characteristics were analyzed and grouped into 12 categories, which we divided into *privacy*, *security*, and *standards and regulations*. Table 1 shows the category descriptions. Some of the characteristics are dependent on others. A complete list of the characteristics analyzed is described in Multimedia Appendix 1.

**Table 1:** Description of the assessed personal health record (PHR) system characteristics.

Category		Description
**Privacy**		
	Privacy policy location	Considers whether user can easily access the privacy policy
	Management and notification of privacy policy changes	Describes whether users are notified of changes in the privacy policy, and the means for doing so
	Access management	Focuses on who shares the information, with whom it is shared, and types of permissions
**Security: confidentiality and integrity**		
	Data management	Considers who manages the information, what information is managed, and where this information comes from
	Data accessed without user’s permission	Describes what data are shared without the user’s explicit consent for secondary use of the data (eg, for marketing, policy)
	Access audit	Informs whether the user can trace with whom his or her information has been shared
	Access criteria	Establishes whether the user is authorized to access the particular resource and what actions she or he is permitted to take with respect to that resource in accordance with certain access criteria
	Authentication	Describes the method used to prevent identity theft
	Without cookies	Indicates whether the system uses cookies
	Safeguards	Presents what security measures are deployed by the PHR system
**Standards and regulations**		
	Standards or regulations	Describes whether the PHR system meets any standards or regulations

Each of these categories satisfied one or more of the eight principles concerning privacy policies by the Canadian Standards Association [21]. The categories, and the principles that they satisfy, are shown in Multimedia Appendix 1.

### Quality Assessment

We evaluated each PHR system in relation to its characteristics. We then assigned three scores to each PHR system: total score (range 0 to 24), security score (range 0 to 14), and privacy score (range 0 to 8). The total score was obtained by adding 1 point for each characteristic that was satisfied. The security and privacy scores were obtained considering only the security and privacy characteristics, respectively, of the categories described above. To address the consistency of the rating system, we used triangulation [26] among the raters—that is, more than one researcher gathered and interpreted the security and privacy characteristics. We used a Cohen kappa coefficient of 0.95, which, according to Landis and Koch [20], indicates almost perfect agreement between two privacy policy assessments performed by two authors. In relation to content validity, we thoroughly reviewed the appropriate scientific literature to find recommendations and standards describing good practices for preparing privacy policies [21-23] to identify the items to be included. Experts then critically reviewed this list for relevance, comprehensibility, completeness, and level of detail.

The test-retest [27] method was used to measure the reliability of the measuring procedure. The same test was performed on the same PHR systems after a month. We obtained a correlation of 0.96 between the scores in the two assessments.

## Results

### Study Selection

**Figure 1:**
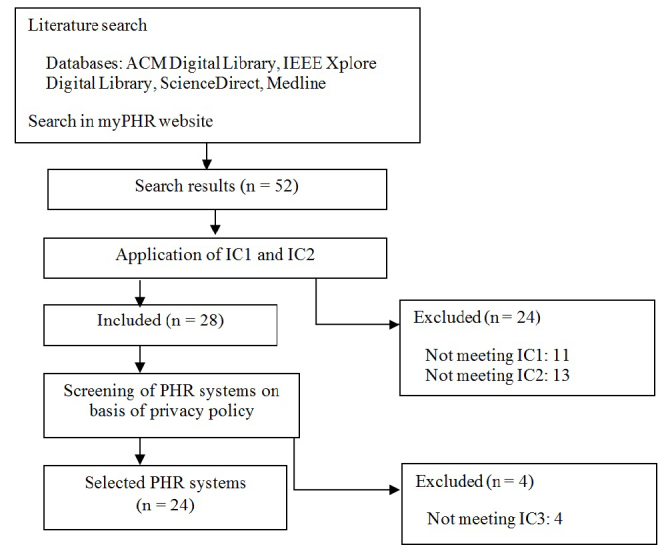
Preferred Reporting Items for Systematic Reviews and Meta-analyses (PRISMA) flow diagram. IC1–3 = inclusion criteria 1 to 3, PHR = Personal Health Record.

We identified 24 PHR systems in the review. The search of databases and the myPHR website provided a total of 52 PHR systems, but we discarded 11 because they did not satisfy IC1 and 13 because the did not satisfy IC2. The privacy policies of the remaining 28 PHR systems were examined, and 4 of these were discarded because they were not patient-centered PHR systems (IC3). Figure 1 shows a PRISMA flow diagram that summarizes this process. The PHR systems included in and discarded from the review are shown in Multimedia Appendix 2.

### Study Characteristics

In this section, we describe the most important features of the PHR systems included in the review. Table 2 shows the percentage of PHR systems that satisfy each characteristic analyzed. Table 3 [28-51] shows the systems selected for the study and the three scores assigned to each: security score, privacy score, and total score. More detailed information about the PHR systems analyzed is provided in the tables shown in Multimedia Appendix 3. The percentages and the scores of the dependent characteristics were calculated in relation to the number of PHR systems that met the nondependent characteristic.

**Table 2:** Evaluation of personal health record (PHR) system characteristics and proportion of PHR systems (n = 24) satisfying each characteristic.

Characteristic		Depends on	n	%
**Privacy policy location**				
	Accessible		23	96
**Notification of changes to privacy policy**				
	Change notification	Accessible	14	61
	Change notification on website	Change notification	12	86
	Change notification directly	Change notification	3	21
**Access management**				
	User grants access		17	71
	User grants access to health care professionals	User grants access	10	59
	User grants access to people with other roles	User grants access	3	18
	Kinds of permissions		5	21
	Access in case of emergency	User grants access	6	35
**Data management**				
	User adds, modifies, removes, and updates information		20	83
	Health care professionals update or add information		5	21
	Family members’ data	User adds, modifies, removes and updates information	3	15
	Connection with other PHRs	User adds, modifies, removes and updates information	4	20
	Monitoring devices		2	8
**Data accessed without user’s permission**				
	Not accessed or information related to the user’s accesses		6	25
**Access audit**				
	Who has accessed it		9	38
	With what aim	Who has accessed it	2	22
**Access criteria**				
	Roles		13	54
	Groups		0	0
	Location		1	4
	Time		2	8
	Transaction type		0	0
**Without cookies**				
	Yes		9	38
**Authentication**				
	Something known		23	96
	Something the user has		1	4
	Biometric factors		0	0
**Safeguards**				
	Physical security measures		15	63
	Limited access		5	21
	Electronic security measures		16	67
	Encrypted data		12	50
	Backup system		4	17
	Defined data security plan		1	4
	Staff training		1	4
	Privacy seal		4	17
**Standard or regulations**				
	HIPAA^a ^considered		10	42
	HIPAA	HIPAA considered	6	60
	HONcode^b^		7	29

^a ^Health Insurance Portability and Accountability Act.

^b ^Health on the Net Foundation Code of Conduct.

**Table 3:** The personal health record (PHR) systems and their assigned scores^a^.

PHR and reference	Security score	Privacy score	Total score
Microsoft HealthVault [28]	14	7	23
Google Health [30]	10	7	18
NoMoreClipBoard [32]	8	6	16
HealthyCircles [34]	11	4	15
myHealthFolders [36]	10	5	15
RememberItNow! [38]	7	8	15
MiVIA [40]	8	4	14
Telemedical [42]	8	4	13
MedicAlert [44]	7	5	12
Juniper Health [46]	8	4	12
MediCompass [48]	6	3	12
myMediConnect [50]	8	3	12
Health Butler [29]	7	3	11
ZebraHealth [31]	8	1	11
My Doclopedia PHR [33]	5	5	11
Dr. I-Net [35]	7	3	11
Keas [37]	5	4	9
MedsFile.com [39]	6	3	9
PatientsLikeMe [41]	2	6	9
My HealtheVet [43]	6	1	9
dLife [45]	3	3	7
MyChart [47]	4	1	7
EMRy Stick [49]	5	2	7
iHealthRecord [51]	4	1	5

^a ^Maximum possible scores: 14 (security score), 8 (privacy score), 24 (total score).

#### Privacy

The privacy policy document must be easily accessible to PHR systems users. This document was accessible or available in 23 of the 24 PHRs, with myMediConnect being the only PHR system to lack this characteristic. The details of its privacy policy were in the website’s FAQ section [50]. Of the PHR systems analyzed, 14 indicated that their users are notified of changes to their privacy policy. Changes could be announced on the home page [28,30,32,33,37,38,40-42,44-46] or via email [39]. A total of 17 of the PHR systems allowed users to grant and revoke access to their data, and 10 indicated that users could grant access to their data to health care professionals. Among these, Google Health and Microsoft HealthVault [28,30] also allowed access to be granted to other system users or to certain services or applications (such as insurance companies or pharmacies).

Only 5 of the PHR systems reviewed defined kinds of permissions. The Google Health PHR system [30] determined two access types for services or applications: write-only access and read/write access. The RememberItNow! PHR system [38] defined three kinds of accesses: write, read, and administrator. Microsoft HealthVault [28] established access levels for users and programs. The Healthy Circles PHR system [34] defined read permission and read/write permission. PatientsLikeMe [41] allowed the contents to be public (anyone could access them) or visible (only PatientsLikeMe users could access them). Finally, only 6 PHRs considered data access in case of an emergency. This access could be total [34] or partial [28].

#### Confidentiality and Integrity

PHRs contain information users’ personal data, which are managed by the user in 20 of the PHRs reviewed. However, MyChart indicated that its users could not manage their own data [47]. Users could only notify the associated health care providers of incorrect data, but not modify them. MyChart was responsible for managing the data. The remainder of the PHR systems did not indicate whether users could manage their data.

A total of 12 PHR systems used aggregated information about users to publish trends or to improve their services [29,30,32-34,37,38,40,41,44,46,48]. Of the PHR systems reviewed, 3 could access users’ identifiable data without their consent [39,45,47].

One mechanism that allowed users to verify whether data confidentiality and integrity were maintained is access audit. In this respect, 9 of the PHR systems permitted users to check who had accessed their data [28,30,32,34,36,38,47,49], and 2 of them allowed users to verify what changes were made [28,30].

PHR systems also presented security measures to maintain data integrity and guarantee confidentiality. Of the PHR systems reviewed, 20 indicated whether they used physical or electronic security measures: 15 of them used physical security measures in their servers. On the other hand, we found 12 PHR systems that used encryption to protect the data during transmission [28,30,32,35,36,38,40,43,46,50], and 4 also stored the data encrypted [35,36,43,46]. And 1, ZebraHealth [31], stated that they regularly reviewed and revised data security plans as required by the evolution of technological and security needs. Some PHR systems even had a privacy seal: Microsoft HealthVault, Healthy Circles, Juniper Health, and dLife were certified by TRUSTe [52].

To avoid unauthorized access of users’ records, an authentication system is required. The most widespread authentication system was the combination of a user ID with a password, which is something the user knows [28-51]. Some PHR systems combined this with the use of an activation code that had been given to users previously [37,47,48,51]. Only 1 PHR used something the user has for authentication. To access MedsFile.com [39], users had to enter the personal identification number on their access card.

As for the access criteria, the most common one was role-based access control [29,31,32,34,36,39,40,42-44,47,48,51]. PHR systems allowed patients, health care providers, insurances, companies, etc, to access records. Access criteria based on location were applied by 1 PHR [40]. This PHR changed the data shown, such as the list of health care providers, depending on the country from which the user accessed the system. Moreover, 2 PHRs enabled users to establish a period of validity for permissions, which were revoked once this period expired [28,38].

#### Standards and Regulations

Some legislation and statements are satisfied by or related to the PHR system analyzed. A total of 6 systems complied with HIPAA, while another 4 indicated in their privacy policy that they were not covered by HIPAA, although some of their procedures were inspired by this regulation. Finally, another 7 PHR systems complied with the Health on the Net Foundation Code of Conduct (HONcode) principles for trustworthy health information. HONcode is the oldest and most-used ethical and trustworthiness code for medical and health-related information available on the Internet.

### Verification of the Information Contained in Privacy Policies

We triangulated [26] sources of data (chosen at random) to raise the level of confidence in our results and to ensure that the data we collected would enable us to draw valid conclusions. Two authors analyzed the privacy aspects of 11 systems by logging in to the PHR systems’ Web portals and verifying whether their privacy policy satisfied the characteristics we had defined. Unfortunately, we were able to verify only a subset of the characteristics analyzed because we could not verify some of them, such as whether the physical measures were really being applied, from the websites. The results obtained were cross-checked against our two initial assessments of the PHR privacy policies. As Table 4 shows, the level of agreement is between high and perfect in three-quarters of the cases [20]. However, the differences we found are not significant because they only lay in some privacy functionalities that were not mentioned in the privacy policies.

**Table 4:** Kappa coefficients for level of agreement in cross-checks of privacy policy assessment.

Personal health record system	Kappa coefficient	Agreement level
Dr. I-Net	0.42	Low
EMRy Stick	0.77	High
HealthButler	0.79	High
HealthyCircles	0.82	Almost perfect
Juniper Health	0.77	High
Microsoft HealthVault	1	Perfect
My DoclopediaPHR	0.9	Almost perfect
myHealthFolders	0.81	Almost perfect
myMediConnect	0.55	Medium
NoMoreClipBoard	0.62	High
RememberItNow!	0.71	High
Telemedical	0.38	Low

## Discussion

The main characteristics of the PHR systems reviewed are summarized below. These characteristics answer our research question of what security and privacy features PRH systems have.

### What Security and Privacy Features do PHR Systems Have?

#### Privacy

In general, most of the PHR systems we reviewed had a document called a privacy policy. This document contains the information related to how the user’s information is managed by the system. The user should be able to access this document [53]. Moreover, users must be notified of changes to the privacy policy, given the importance of this document. To fulfill this requirement, most of the PHR systems published an advertisement on their website, which obliges the user to check the PHR website to verify whether the privacy policy has changed. Some authors [54] believe that direct notification of any change is a better solution. One characteristic not found in the PHRs we reviewed is that of notifying users when their data have been exposed. Some regulations, such as the Directive on Privacy and Electronic Communications of the European Union [55], indicate that users have the right to be notified of any personal information disclosure. Most states in the United States also have data breach notification laws [56], which PHR systems must satisfy. These require a data custodian to report a data breach to the individuals affected, state attorneys general, the media, consumer reporting agencies, or other government agencies. One means to ensure that users trust their data security is to indicate that the PHR website is certified by a certification authority. The PHR systems we reviewed used the TRUSTe [52] certification, which guarantees that the security requirements included in the privacy policies are supported by the website.

With regard to PHR access management, 71% of PHR systems allowed users to grant and revoke access to their data. This characteristic is particularly important because users require more flexible ways of sharing data, allowing the user to choose who can access their data, which data they can access, and at what level of access [57]. A problematic issue is the access to users’ data in case of emergency—that is, when users cannot explicitly grant access. We found that 35% of PHRs considered this case and provided some type of mechanism to permit the appropriate health care professionals (previously authorized by the user) to access the user’s data. Some PHR systems, such as Microsoft HealthVault, allowed users to select what information could be shared and with whom in case of emergency. Nevertheless, emergency access increases the risk of data breaches. Some national laws assume implicit patient consent in an emergency situation [58], which does not guarantee the privacy of patients’ data. Moreover, this unusual access adds an extra complexity level to the access control model [58]. On the other hand, not all users are very inclined to share their data in a health emergency. Users with good or excellent health are less likely to share their data during this kind of situation [59].

Finally, ownership of the PHR is an important issue to consider. In Europe, although the PHR can store patient information from a health care provider, the patient owns only the copy stored in the PHR, not the information stored with the provider [60]. This is, for instance, the case in the Dutch system. Such a system allows users to remove data from their PHR, but they cannot remove data from a hospital EHR. Other approaches allowed users to access but not modify their PHR, such as HealthSpace [57]. This may make the PHR of less value to patients and physicians, as no information flows back, but it does provide more security. In the United States, there is the case of My HealtheVet, which is a PHR system developed by the Department of Veterans Affairs. According My HealtheVet’s privacy policy, although the content is managed by the Department, the PHR is the property of the veteran and she or he can also manage the information [61].

#### Confidentiality and Integrity

We examined patient-centered PHR systems in this review, and they allow users to manage their data. In other words, users can add, modify, remove, and update their health data in 83% of cases, according to our review. Connecting the PHR to the EHR would lead to more comprehensive data management by patients [62]. However some physicians have expressed their concern about giving patients so much control over their records, because the information stored in PHRs might be less accurate if patients do not know what exactly is included in them, in comparison with non-patient-centered PHRs [63]. Moreover, if a PHR is hacked—and the patient’s data are modified—then, physicians cannot be sure of the correctness of the data [63]. When information comes from several sources, greater privacy and security risks emerge. However, determining the most appropriate strategy remains an open question: to have multiple reliable sources of information, or to have the patient be the only information source.

Few PHRs permit users to check who accessed their data. This aspect should be improved because, according to HIPAA’s Privacy Rule and Security Rule and to ISO 13606, users should be aware of how their information has been shared.

We found that 3 (13%) of PHR systems used information related to users’ accesses and identified user information to monitor system use without the user’s explicit consent. Since the users’ privacy should be guaranteed, their identifiable information should not be accessed without their consent [64]. Half of the PHR systems used de-identified or aggregated user information. However, it is very difficult to retrieve sufficient information when aggregated data are used in order to ensure that patients cannot be identified, so some risk of re-identification will usually remain [8,65]. A further issue is that PHR privacy policies did not indicate what information they aggregated. PHR designers could consider studies such as that of Sweeney, who designed a model called k-anonymity, and the accompanying policies that allow the individual’s information to be protected, because this cannot be distinguished from, at least, k - 1 other individuals’ information [66]. With regard to the information de-identification process, HIPAA indicates that there are two ways to do this: a formal determination by a qualified statistician, or the removal of specified identifiers of the individual and of the individual’s relatives, household members, and employers. Removal of identifiers is adequate only if the entity covered has no actual knowledge that the remaining information could be used to identify the individual. In any case, one of these two means is required [2].

The PHR systems must take physical and electronic measures to protect user information [67]. Of the PHR systems we analyzed, in their privacy policies, 63% indicated their physical measures and 67% explicitly stated their electronic security measures; however, only 4 (17%) stated that the data were encrypted both for transmission over the network and for storage. The most widely used encryption scheme for communications was secure socket layer. However, encryption is only part of the solution to protect data. There are also other threats, such as virus-infected systems, against which the PHR systems must be protected. Although there are no well-documented examples of EHR/PHR systems linked to security breaches [68], designers should consider threats to Web applications at least when they deploy their PHR system. In 2008, over 63% of all documented vulnerabilities affected Web applications [69].

Important for security vulnerability is authentication [70]. All the PHR systems we analyzed used only one authentication method, the use of something the user knows or has. However, two of the following three methods are recommended for inclusion in an identification system: something a person knows, such as login ID; something a person has, such as an access card; or something that identifies a person, such as biometrics. Therefore, designers should incorporate another authentication system to strengthen authentication [71]. Moreover, the use of passwords as an authentication mechanism is exposed to multiple types of attacks, such as electronic monitoring of network traffic to capture information, or unauthorized access to the password file.

Finally, 38% of the PHR systems used cookies to remember that the user had already logged in. Using cookies increases the likelihood of identity attacks because the cookie’s authentication data can be intercepted by a hacker to gain access to the user’s health data [70].

#### Standards and Regulations

Finally, less than half of the PHR systems we reviewed were based on standards or regulations, and this shows that there is no guarantee that the privacy and security of patients’ data is ensured. The most frequently referenced regulation is HIPAA, used in the United States. HIPAA is a federal law that protects health information and ensures that patients have access to their own medical records while assigning new responsibilities to those in charge of protecting this information. Although PHR systems are not required to meet HIPAA by law, users might believe that their data are better protected if the PHR satisfies HIPAA [72].

### Limitations

This study had several limitations. Although we conducted a comprehensive literature search on numerous databases using a variety of pertinent search terms, certain PHR systems may have been overlooked due to the lack of indexing in the searched databases. In addition, we recognize that several key PHR systems that were included in the original sample of 51 were excluded as a result of selection criteria. Moreover, we may have excluded some PHR systems if we did not find their privacy policies on their website.

Since this study only analyzed the security and privacy characteristics of PHR systems, it lacks information about the users. Our results cannot easily be generalized to populations, since PHR systems are not equally used by people of different age groups.

The scope of this study did not include analysis of real functionality of PHR systems, and some PHR systems may not satisfy their own privacy policies, so incorrect data may have affected the results of the study. However, this limitation is diminished because we cross-checked the results against an evaluation of actual functionality of 50% of the PHRs.

Another limitation of our study is related to third-party access to the PHR. This characteristic turns PHR systems into a more flexible tool, although it would be necessary to analyze the privacy policies of these parties.

### Conclusions

In general, PHR systems allow users to manage their personal health data and to control who has access to them. However, there is a debate regarding the degree to which individuals should be able to control this access, and the forms that this control may take: some PHR systems allow their users only read-only access, while others offer individuals total control [73,74].

The strengths and weaknesses in the privacy and security of PHR systems will be useful for PHR users, health care professionals, decision makers, and system builders. In accordance with the privacy policies, PHR systems do not provide an in-depth description of the security measures used. The designs of privacy policies also need to be improved to include more detailed information related to security measures, and PHR system designers should focus their efforts on increasing the quality of security measures at all stages of system development [75].

The use of standards and regulations by PHR systems is still low. The majority of companies that design PHR systems are not covered by HIPAA [7]. This may be one of the reasons why users do not use PHR systems [72].

Finally, the development of third-party applications that add new functionality to PHR systems is increasing. An example of this is Microsoft HealthVault, which has more than 50 third-party applications [28]. This connection to other applications, such as PHR systems, could also cause important security breaches.

## Multimedia Appendix 1

Characteristics analyzed and principles that they satisfy.

## Multimedia Appendix 2

List of personal health record systems excluded and included in the study.

## Multimedia Appendix 3

Characteristics of personal health record systems included in the review.

## Abbreviations

EHR: electronic health record
HIPAA: Health Insurance Portability and Accountability Act
HONcode: Health on the Net Foundation Code of Conduct
ISO: International Organization for Standardization
PHR: personal health record
PRISMA: Preferred Reporting Items for Systematic Reviews and Meta-analyses
