# Supplementary material for: Are Personal Health Records Safe? A Review of Free Web-Accessible Personal Health Record Privacy Policies
Source: J Med Internet Res. 2012 Aug 23;14(4):e114. doi: 10.2196/jmir.1904 (PMC3510685; doi:10.2196/jmir.1904)
Supplement: Supplementary file 1 [file jmir_v14i4e114_app1.pdf]

Multimedia Appendix 1. Characteristics analyzed and principles which are satisfied by them

1. Privacy Policy Location (PPL). This category considers whether the Privacy Policy can be easily accessed by the user, and contains the following characteristics:
    1.1. Accessible (PPL1). The PHR has an accessible Privacy Policy document
2. Changes Management/Notification in Privacy Policy (CPP). This category describes whether the changes in the Privacy Policy are notified to the users, and the means used to do so.
    2.1. Changes notified (CPP1). The users are notified of changes to the Privacy Policy (This characteristic can only be satisfied if PPL1 is also satisfied)
    2.2. Changes notified on website (CPP2). The PHR announces the changes to its Privacy Policy on the website. (This characteristic can only be satisfied if CPP1 is also satisfied)
    2.3. Changes notified directly (CPP3). The changes to the Privacy Policy are notified by sending the users a direct notification. (This characteristic can only be satisfied if CPP1 is also satisfied)
3. Access Management (ASM). This category describes who shares the information, with whom it is shared, and types of permissions
    3.1. Users grant access (ASM1). The users grant and revoke access to their data
    3.2. Users grant access to healthcare professionals (ASM2). The users can grant healthcare professionals access to their data. (This characteristic can only be satisfied if ASM1 is also satisfied)
    3.3. Users grant access to people with other roles (ASM3). The users can grant certain services (such as insurance companies or pharmacies), friends, family, or applications access to their data. S/he can indicate what data are accessed by an individual or a set of individuals with common characteristics (roles) (This characteristic can only be satisfied if ASM1 is also satisfied)
    3.4. Kinds of permissions (ASM4). The PHR defines various kinds of permissions

3.5. Access in case of emergency (ASM5). The PHR considers data access in the case of an emergency. (This characteristic can only be satisfied if ASM1 is also satisfied)

4. Data Management (DM). This category describes who manages the information, what information is managed and where this information comes from

4.1. User adds, modifies, removes and update information (DM1). The information related to users' personal and health data are managed by the user. The users can add, modify, remove and update their health data

4.2. Healthcare professionals update or add information (DM2). Healthcare professionals are authorized to send users' health data to the users' accounts

4.3. Family members' data (DM3). The users manage their health data and their family members' data (This characteristic can only be satisfied if DM1 is also satisfied)

4.4. Connection with other PHRs (DM4). The users can transfer their information from other PHRs. (This characteristic can only be satisfied if DM1 is also satisfied)

4.5. Monitoring devices (DM5). The users' data are obtained from monitoring devices connected to their mobile phones or PCs

5. Data accessed without the user's permission (DA). This category describes what data are shared without the user's explicit consent

5.1. Not accessed or information related to the users' accesses (DA1). The PHR uses information related to the users' accesses in order to monitor and to analyze the system use without the users' explicit permission, or it does not access users' data without their explicit consent for secondary use of the data (e.g., marketing, policy, etc.)

6. Access Audit (AA). This category describes whether the user can see with whom their information has been shared

6.1. Who has accessed (AA1). The PHR system permits the users to check who has accessed their data

6.2. With what aim (AA2). The PHR system allows the users to see who has accessed their data and with what aim. The user can check if data accessed was read, modified or removed (This characteristic can only be satisfied if AA1 is

also satisfied)

7. Access criteria (AC). The system must establish whether the user is authorized to access the particular resource and what actions s/he is permitted to perform on that resource in accordance with certain access criteria
   7.1. Roles (AC1). Using roles is an efficient means to assign rights to a type of user who performs a certain task. The role is based on a job assignment or function
   7.2. Groups (AC2). If several users require the same type of access to information and resources, they are placed in a group
   7.3. Location (AC3). Physical or logical location can also be used to restrict access to resources
   7.4. Time (AC4). Access is is permitted only for a finite period of time
   7.5. Transaction type (AC5). Transaction-type restrictions can be used to control what data is accessed in certain types of functions and what commands can be carried out on the data
8. Without Cookies (C). This category describes whether the system is a system without cookies
   8.1. Yes (C1). The PHR is a system without cookies
9. Authentication (AU). Method used to prove that the user is who s/he says s/he is
   9.1. Something known (AU1). User is provided with something which s/he knows (a password, PIN, etc.)
   9.2. Something that the user has (AU2). User is provided with something which s/he has (a key, an access card, a badge, etc.)
   9.3. Biometric factors (AU3). User is provided with something which s/he is (physical attributes)
10. Safeguards (SS). This category describes security measures deployed by the PHR system
    10.1. Physical security measures (SS1). The PHR uses physical security measures in its servers
    10.2. Access limited (SS2). The PHR has computer servers whose access is limited to a small number of people
    10.3. Electronic security measures (SS3). The PHR encrypts the users’ data when it is stored and transmitted

10.4. Encrypted data (SS4). The PHR manages encrypted data

10.5. Back-up system (SS5). The PHR uses back-up systems as an extra security measure

10.6. Data security plan defined (SS6). Data security plans have been defined in the PHR system. They are regularly reviewed and revised as technological and security needs change

10.7. Staff training (SS7). The staff receives training on the latest security technology available in the PHR system

10.8. Privacy Seal (SS8). The PHR website has obtained certification from a trusted third-party authority, for example, TRUSTe. This certification indicates that the site has been self-certified as complying with the site's own privacy statement.

11. Standard or regulations (ST). This category describes whether the PHR meets any standards

11.1. HIPAA considered (ST1). HIPAA was considered in the design of Privacy Policy, but the company which made the PHR is not an entity covered by HIPAA.

11.2. HIPAA (ST2). The PHR is in compliance with HIPAA (This characteristic can only be satisfied if ST1 is also satisfied)

11.3. HONcode (ST3). The PHR complies with the (Health On the Net Code of Conduct) HONcode principles. HONcode is "the oldest and the most used ethical and trustworthy code for medical and health related information available on Internet".

Table 5. Categories of characteristics of Privacy, Standards and Regulations extracted and principles which are satisfied by them

Principle	Category			
Privacy, Standards and Regultations				
	Privacy Policiy Location	Access Management	Changes in Privacy Policy	Standards and Regulations
**Consent**		X		
**Limiting collection**				
**Limiting use, disclosures, and retention**		X		
**Accuracy**				
**Safeguards**				

**Openness**	X		X	
**Individual Access**		X		
**Standards**				X

Table 6. Categories of characteristics of Security extracted and principles which are satisfied by them

**Principle**	**Category**						
**Security**							
	**Data Management**	**Access Audit**	**Access Criteria**	**Data accessed without the user's permission**	**Cookies**	**Authentication**	**Safeguards**
**Consent**				X			
**Limiting collection**				X			
**Limiting use, disclosures, and retention**		X		X			
**Accuracy**	X						
**Safeguards**			X		X	X	X
**Openness**							
**Individual Access**	X	X					
**Standards**							
