# Supplementary material for: Are Personal Health Records Safe? A Review of Free Web-Accessible Personal Health Record Privacy Policies
Source: J Med Internet Res. 2012 Aug 23;14(4):e114. doi: 10.2196/jmir.1904 (PMC3510685; doi:10.2196/jmir.1904)
Supplement: Supplementary file 2 [file jmir_v14i4e114_app2.pdf]

Multimedia Appendix 2. List of PHRs excluded and included in the study

Table 7: List of PHRs included and discarded in the review

	PHR	Source	Discard Criteria
**1**	Medefile	ScienceDirect	IC1
**2**	RelayHealth	ScienceDirect, ACM Digital Library	IC1
**3**	LifeSensor	ScienceDirect	IC1
**4**	IQHealth	ScienceDirect	IC1
**5**	CapMed icePHR Mobile	ScienceDirect, ACM Digital Library	IC1
**6**	MyMedicalRecords.com	ScienceDirect	IC1
**7**	SecureMed	Medline	IC1
**8**	My Health	ScienceDirect	IC1
**9**	Dossia	ScienceDirect, Medline, ACM Digital Library	IC1
**10**	Lynxcare	ScienceDirect	IC1
**11**	AboutMyHealth	ScienceDirect	IC3
**12**	MyMediList	myPHR.com	IC3
**13**	ItRunsInMyFamily	myPHR.com	IC3
**14**	WorldMedcard	ACM Digital Library	IC3
**15**	Patient Power	myPHR.com	IC1
**16**	Compiling Your Family Health History	myPHR.com	IC2
**17**	Powerful Patient Solutions, Inc.	myPHR.com	IC2
**18**	MyPRO Medical-Health Records Organizer	myPHR.com	IC2
**19**	Jakoter Health Organizer	myPHR.com	IC2
**20**	HealtheTracks	myPHR.com	IC2
**21**	So Tell Me Medical Organizer	myPHR.com	IC2
**22**	Organized Solutions	myPHR.com	IC2
**23**	My Vital Data	myPHR.com	IC2
**24**	People Chart	myPHR.com	IC2
**25**	HealthNote	myPHR.com	IC2
**26**	My Personal Guardian	myPHR.com	IC2
**27**	Personal Medical Records	myPHR.com	IC2

**28**	Merck Source	ScienceDirect	IC2
**29**	VIA	myPHR.com	Included
**30**	My Doclopedia PHR	ACM Digital Library, myPHR.com	Included
**31**	Dr. I-Net	ACM Digital Library, myPHR.com	Included
**32**	EMRy STICK	ScienceDirect, ACM Digital Library, myPHR.com	Included
**33**	Google Health	ScienceDirect, MedLine, ACM Digital Library, IEEE Digital Library, myPHR.com	Included
**34**	HealthButler	ACM Digital Library	Included
**35**	Keas	ACM Digital Library, myPHR.com	Included
**36**	dLife	ACM Digital Library, myPHR.com	Included
**37**	iHealthRecord	ACM Digital Library, myPHR.com	Included
**38**	myMediConnect	ACM Digital Library, myPHR.com	Included
**39**	MedicAlert	ScienceDirect, ACM Digital Library, myPHR.com	Included
**40**	MedsFile.com	ACM Digital Library, myPHR.com	Included
**41**	Microsofth HealthVault	ScienceDirect, Medline, ACM Digital Library, IEEE Digital Library, myPHR.com	Included
**42**	MyChart	ScienceDirect, Medline, ACM Digital Library, myPHR.com	Included
**43**	myHealthFolders	ScienceDirect, ACM Digital Library, myPHR.com	Included

**44**	NoMoreClipBoard.com	ACM Digital Library, myPHR.com	Included
**45**	RememberItNow!	ACM Digital Library, myPHR.com	Included
**46**	Telemedical.com	ScienceDirect, ACM Digital Library, myPHR.com	Included
**47**	Juniper Health	ACM Digital Library, myPHR.com	Included
**48**	My HealtheVet	ScienceDirect, Medline	Included
**49**	ZebraHealth	ACM Digital Library, myPHR.com	Included
**50**	MediCompass	ACM Digital Library, myPHR.com	Included
**51**	PatientsLikeMe	ScienceDirect, Medline, ACM Digital Library	Included
