# Supplementary material for: Are Personal Health Records Safe? A Review of Free Web-Accessible Personal Health Record Privacy Policies
Source: J Med Internet Res. 2012 Aug 23;14(4):e114. doi: 10.2196/jmir.1904 (PMC3510685; doi:10.2196/jmir.1904)
Supplement: Supplementary file 3 [file jmir_v14i4e114_app3.pdf]

Multimedia Appendix 3. Characteristics of PHRs included in the review

Table 8: Summary of the Assessment of the Privacy Policy characteristics in PHR Systems

PHR	Privacy Policy Location	Changes in Privacy Policy
**Microsoft Health Vault [28]**	Accessible	Changes will be notified on the website
**HealthButler [29]**	Accessible	Changes not notified
**Google Health [30]**	Accessible	The changes are announced on the home page
**ZebraHealth [31]**	Accessible	Changes not notified
**NoMoreClipBoard.com [32]**	Accessible	Changes will be notified by posting a notice on the Website
**My Doclopedia PHR [33]**	Accessible	Changes will be posted on the Site
**Healthy Circles [34]**	Accessible	Changes not notified
**Dr. I-Net [35]**	Accessible	Not indicated
**myHealthFolders [36]**	Accessible	Users are notified of any changes
**Keas [37]**	Accessible	Changes will be notified by placing a prominent notice on the Keas home page or by sending users a direct notification
**RememberItNow! [38]**	Accessible	User will be notified of changes by email or by a notice posted on the home page of the Site
**MedsFile.com [39]**	Accessible	Any changes will be communicated to users via email at least three (3) days in advance of the effective date
**VIA [40]**	Accessible	Changes will be posted on its home page
**PatientsLikeMe[41]**	Accessible	Changes will be posted on the home page
**Telemedical.com [42]**	Accessible	The changes will be published on the home page
**My HealtheVet [43]**	Accessible	Not indicated
**MedicAlert [44]**	Accessible	Changes will be posted on the website
**dLife [45]**	Accessible	Changes will be posted on the website
**Juniper Health [46]**	Accessible	Changes will be posted on the home page
**MyChart [47]**	Accessible	Not indicated
**MediCompass [48]**	Accessible	Not indicated
**EMRy STICK [49]**	Accessible	Not indicated

**myMediConnect; Passport MD [50]**	Not visible	Not indicated
**iHealthRecord [51]**	Accessible	Changes not notified

Table 9: Summary of the Assessment of the Access Management in PHR Systems

**PHR**	**Access Management**
**Microsoft Health Vault [28]**	Users can share their data with friends, family, healthcare professionals and programs. Access Level for users: View-only access, View-and-modify access and Custodian access. Access Level for programs: view, add and modify. Emergency profiles can be defined by the user.
**HealthButler [29]**	The user grants healthcare professionals access to their health data
**Google Health [30]**	The users grant/revoke access to their data to other system users or certain services (insurance companies, healthcare providers, pharmacies). Kinds of access for services are: write-only access and read/write access
**ZebraHealth [31]**	Not indicated
**NoMoreClipBoard.com [32]**	The users grant healthcare professionals access to their data and their family data. Users can create an emergency access PIN that can allow someone, typically an emergency responder, to see selected information in a view-only mode
**My Doclopedia PHR [33]**	The users grant healthcare professionals access to their data
**Healthy Circles [34]**	The user grants healthcare professionals access to their health data. Types of permissions: read and read/write.
**Dr. I-Net [35]**	Users (patients) have to authorize healthcare professionals to use their health data
**myHealthFolders [36]**	The users grant/revoke access to their data to BJC healthcare professionals. Healthcare professionals with the user’s emergency card number can access their data
**Keas [37]**	Not indicated
**RememberItNow! [38]**	Users can share their health information with family, friends, and health care professionals. Users can grant write, read or administrator access
**MedsFile.com [39]**	Users cannot grant access to their data
**VIA [40]**	The information can only be shared with the users’ permission

**PatientsLikeMe[41]**	Users can share their health information with other PatientsLikeMe users (visible access) or everybody (public access). PatientsLikeMe may use a user's data in the case of an emergency.
**Telemedical.com [42]**	The users grant/revoke access to their data
**My HealtheVet [43]**	Users' information cannot be accessed by Veterans' Affairs doctors or nurses
**MedicAlert [44]**	In the event of an emergency, users are authorized to release all medical and other confidential information to the appropriate emergency responders. Otherwise, users explicitly grant access to their data
**dLife [45]**	Not indicated
**Juniper Health [46]**	Users choose with whom to share their information
**MyChart [47]**	Users can grant access to their data only to health care professionals of MyChart
**MediCompass [48]**	The users grant/revoke access to their data to healthcare professionals registered to use MediCompass
**EMRy STICK [49]**	Users control who can access their personal health information
**myMediConnect; Passport MD [50]**	Users grant healthcare professionals access to their data. Healthcare professionals can view the users' data in the case of an emergency
**iHealthRecord [51]**	Not indicated

Table 10: Summary of the Assessment of the Data Management in PHR Systems

**PHR**	**Data Management**
**Microsoft Health Vault [28]**	Users may add, modify and delete their data. The system is connected with other PHRs and healthcare devices
**HealthButler [29]**	The users introduce their health data. Individuals registered with Health Butler have the option of linking to Google Health
**Google Health [30]**	By default, the user is the only one who can manage their data
**ZebraHealth [31]**	Users add their information
**NoMoreClipBoard.com [32]**	The users update their health data. Authorized healthcare professionals can send users' health data to their accounts
**My Doclopedia PHR [33]**	Users can review and update any personal information that the system has already collected
**Healthy Circles [34]**	The users introduce their health data. Users may access health-related information about themselves that is stored with third party-data services such as Microsoft HealthVault or Google Health. Health-related information about the users received from healthcare professionals. The users can modify, add, or delete their data.

**Dr. I-Net [35]**	Users can add to and modify their health records, but medical history, lab results and specialty charts will have to be filled in by the appropriate medical personnel
**myHealthFolders [36]**	The user is the only one who can modify, update and delete their data and their family data
**Keas [37]**	Users choose what information to put in their records. Users can transfer their health information from their Google Health or Microsoft HealthVault account to their Keas account
**RememberItNow! [38]**	The user is the only person who can modify, update and delete their data
**MedsFile.com [39]**	Users can update, correct and delete their information
**VIA [40]**	The information stored on MiVIA belongs to the user
**PatiensLikeMe [41]**	Users can update, correct and delete their information
**Telemedical.com [42]**	The user is the only person who can modify, update and delete their data and their family data
**My HealtheVet [43]**	Users' add, modify and delete their information
**MedicAlert [44]**	Users may review, update and modify their personal medical information
**dLife [45]**	Not indicated
**Juniper Health [46]**	Users choose what information to put in their records. Users can create health records for themselves and their family members
**MyChart [47]**	Users can notify data failure
**MediCompass [48]**	Data are provided by many self-monitoring devices connected to the user's phone or PC
**EMRy STICK [49]**	The user is the only person who can modify, update and delete their data
**myMediConnect; Passport MD [50]**	The users' health data may be obtained from their healthcare providers (by paying) or from the users (free). System manages users' data (paying) or the user manages them (free). System is connected to Microsoft HealthVault
**iHealthRecord [51]**	Not indicated

Table 11: Summary of the Assessment of the Data Access in PHR Systems

PHR	Data accessed without the user's permission
**Microsoft Health Vault [28]**	They may collect information about users' visits, including the pages users view, the links users click, and other actions taken in connection with the Service
**HealthButler [29]**	Health Butler compiles aggregate data without identifying the user individually
**Google Health [30]**	Information related to the accesses (number of sign-ins and clicks). Google use de-identified user information to publish trends

**ZebraHealth [31]**	ZebraHealth may use third party products to track usage of its applications and store information such as the domain name from which users contact it, the pages users request, the site referred to, and the time users spend on its site
**NoMoreClipBoard.com [32]**	The system may collect and summarize non-personal information for internal use
**My Doclopedia PHR [33]**	IP (Internet Protocol) address, domain server, type of computer and type of web browser are collected to improve the system. This system shares users' data with advertisers and other third parties on an aggregate basis
**Healthy Circles [34]**	Their servers may also automatically collect information, such as IP addresses, browser type, pages visited, access times, duration of visit, referring URL, platform, links accessed, timestamp and exit page information ("Automated Information") with regard to each visitor to the Site, whether or not such a visitor is a logged-on user. They may disclose aggregated, non-personal data generated from the Service to third parties.
**Dr. I-Net [35]**	Not indicated
**myHealthFolders [36]**	Information related to the accesses: IP address, browser type, date and time
**Keas [37]**	De-identified and/or aggregated information may be used/gathered to improve the services provided through Keas
**RememberItNow! [38]**	They may collect certain information from visitors to the Site, such as Internet addresses. They may also track and analyze individual usage and volume statistical information ("Aggregated Information") from their visitors and customers. Non-identifiable anonymous data may be disclosed to third parties
**MedsFile.com [39]**	IP addresses, browsers, pages viewed, number of visitors, services purchased are collected to improve the site. They use users' personally-identifying information to improve their marketing and promotional efforts, to statistically analyze site usage, to improve their content and product offerings and to customize their site's content, layout, and services
**VIA [40]**	The system uses IP address and aggregate demographic information to analyze trends
**PatiensLikeMe [41]**	They can provide their partners with anonymized, aggregated community data with the goal of increasing involvement in disease research
**Telemedical.com [42]**	Not indicated
**My HealtheVet [43]**	System automatically collects information about users' visits (IP address, operating system, browser)
**MedicAlert [44]**	MedicAlert Foundation may release de-indentified member information for valid medical research purposes or to provide additional services

**dLife [45]**	They may share the users' identifiable data with third parties for direct marketing and other purposes, but users may decline to share their data for direct marketing. The system monitors the IP address, URL and time of accesses
**Juniper Health [46]**	Not indicated
**MyChart [47]**	They may use and disclose PHI (Protected Health Information) about users without their written consent: for treatment, for payment, for health care operations, family members and friends, appointments, hospital directory, fundraising activities, public health and government functions, organ, eye and tissue donation, research
**MediCompass [48]**	The system may share non-personal, non-identifiable, summary, and/or aggregate data with its partners and other third parties
**EMRy STICK [49]**	EMRy Stick's servers automatically record log information about users' use of EMRy Stick Health (number of sign-ins and number of times a link was clicked). This information is temporarily stored in association with users' account for two weeks, at which point it is aggregated with other data and is no longer associated with the account
**myMediConnect; Passport MD [50]**	Not indicated
**iHealthRecord [51]**	The system uses IP address and user's domain name for internal review

---

Table 12: Summary of the Assessment of the Access Audit in PHR Systems

**PHR**	**Access Audit**
**Microsoft Health Vault [28]**	Users can see who has accessed their data and why
**HealthButler [29]**	Not indicated
**Google Health [30]**	The users can see who has accessed the information, and with what aim
**ZebraHealth [31]**	Not indicated
**NoMoreClipBoard.com [32]**	Users are notified of accesses with emergency PIN
**My Doclopedia PHR [33]**	Not indicated
**Healthy Circles [34]**	The user will be able to receive a list of individuals or entities to whom s/he has or had granted access or who have otherwise received disclosures of their personal information or personal health information.
**Dr. I-Net [35]**	Not indicated

**myHealthFolders [36]**	Users are notified whenever their accounts are accessed
**Keas [37]**	Not indicated
**RememberItNow! [38]**	Users can view a list of who has access to their information
**MedsFile.com [39]**	Users will be notified at the time of data collection or transfer if their personally-identifying information will be shared with a third party
**VIA [40]**	Not indicated
**PatiensLikeMe [41]**	Not indicated
**Telemedical.com [42]**	Not indicated
**My HealtheVet [43]**	Not indicated
**MedicAlert [44]**	Not indicated
**dLife [45]**	Not indicated
**Juniper Health [46]**	
**MyChart [47]**	Users have the right to request a list of instances in which the system has disclosed their PHI
**MediCompass [48]**	Not indicated
**EMRy STICK [49]**	Users can view a list of who has access to their data
**myMediConnect; Passport MD [50]**	Not indicated
**iHealthRecord [51]**	Not indicated

Table 13: Summary of the Assessment of methods of Authentication and Authorization and Cookies in PHR Systems

**PHR**	**Access Criteria**	**Authentication**	**Cookies**
**Microsoft Health Vault [28]**	Access time	User and password	Yes
**HealthButler [29]**	User role	User and password	Not indicated
**Google Health [30]**	Not indicated	User and password	Yes
**ZebraHealth [31]**	User role	User and password	Yes
**NoMoreClipBoard.com [32]**	User role	User and password	Not indicated
**My Doclopedia PHR [33]**	Not indicated	User and password	Not indicated
**Healthy Circles [34]**	User role	User and password	Yes

**Dr. I-Net [35]**	Not indicated	User and password	Not indicated
**myHealthFolders [36]**	User role	User and password	Yes
**Keas [37]**	Not indicated	User and password	Yes
**RememberItNow! [38]**	Access time	User and password	Yes
**MedsFile.com [39]**	User role	Smart card	Yes
**VIA [40]**	User role and location	User and password	Not indicated
**PatiensLikeMe [41]**	Not indicated	User and password	Session and Persistent Cookies
**Telemedical.com [42]**	User role	User and password	Not indicated
**My HealtheVet [43]**	User role	User and password	Yes, not persistent
**MedicAlert [44]**	User role	User and password	Yes, session cookies
**dLife [45]**	Not indicated	User and password	Yes
**Juniper Health [46]**	Not indicated	User and password	Yes
**MyChart [47]**	User role	User and password	Not indicated
**MediCompass [48]**	User role	User and password	Yes, session cookies
**EMRy STICK [49]**	Not indicated	User and password	Not indicated
**myMediConnect; Passport MD [50]**	Not indicated	User and password	Not indicated
**iHealthRecord [51]**	User role	User and password	Yes, session cookies

---

Table 14: Summary of the Assessment of Security Measures of PHR Systems.

PHR	Security
**Microsoft Health Vault [28]**	Microsoft stores the users' personal information in computer servers which are located in controlled facilities and with limited access. The Service sends all communications, using SSL encryption
**HealthButler [29]**	Not indicated
**Google Health [30]**	Physical security measures for servers. Data are encrypted using SSL and firewalls. Google use back-up systems. Access limited to a small number of people

**ZebraHealth [31]**	ZebraHealth has implemented appropriate physical, electronic, procedural and managerial safeguards to protect all information that is submitted or transmitted to their site. They maintain multiple copies of backup data at their physical location as well as securely off-site. Their data security plans are reviewed regularly and revised as technological and security needs change. They use current cryptography technology to protect data during transmission and storage when necessary. They employ multi-factor authentication to protect against unauthorized access to users' data. They are active in preventing misuse, disclosure, alteration and loss of users' personal data
**NoMoreClipBoard.com [32]**	This system uses SSL. The site security is approved by VerisignTM
**My Doclopedia PHR [33]**	Users' personal information is transmitted via secured and encrypted channels
**Healthy Circles [34]**	They maintain physical, electronic and procedural safeguards that are designed to comply with applicable laws and to keep personal information and the users' health-related data safe.
**Dr. I-Net [35]**	Data stored are encrypted using SSL Verisign encryption. Passwords are also encrypted. This site has security measures in place to protect against the loss, misuse, or alteration of the information stored in its database
**myHealthFolders [36]**	Data are stored encrypted in secure servers which are in secure places. Data are transmitted using 128 SSL encryption scheme
**Keas [37]**	The personal information is encrypted in computer servers with limited access that are located in controlled facilities
**RememberItNow! [38]**	The system uses SSL encryption to ensure that users' sensitive information is protected and encrypted when processing credit card transactions. Data are stored in secure servers that use a firewall
**MedsFile.com [39]**	They employ reasonable and current security methods and they maintain physical, electronic and procedural safeguards to prevent unauthorized access, maintain data accuracy, and ensure correct use of information. They limit access to the information to employees and service and equipment providers
**VIA [40]**	Relevant contact information is stored in secure servers that are protected 24/7. The data are encrypted by SSL
**PatientsLikeMe [41]**	Not indicated
**Telemedical.com [42]**	The medical record information is secured by SSL and Digital Certificate encryption and authentication technology
**My HealtheVet [43]**	Users' identifiable information is stored and transmitted, encrypted by using SSL

**MedicAlert [44]**	All electronic data is securely encrypted and backed up in industrially safe locations (earthquake proof, fireproof, flood proof). Security is reviewed by a third party, and staff receives training on the latest security technology available
**dLife [45]**	They take commercially reasonable precautions to safeguard personally identifiable information provided to them, but they cannot guarantee that such information will not be lost, disclosed or accessed by accidental circumstances or by the unauthorized acts of others
**Juniper Health [46]**	The system stores the users' personal information encrypted in computer servers which are located in controlled facilities and with limited access. Sensitive information is encrypted using SSL
**MyChart [47]**	Not indicated
**MediCompass [48]**	The system uses multiple levels of data security, including data encryption (128-SSL), database field encryption, and physical server site security (with 24-hour guards). They use video surveillance cameras, motion, temperature, and vibration detectors
**EMRy STICK [49]**	Not indicated
**myMediConnect; Passport MD [50]**	Data are encrypted by using 256-bit SSL. All information is kept in a highly secure USB-based facility, guarded 24 hours a day by armed guards, security sensors, cameras, and multiple levels of security measures
**iHealthRecord [51]**	Medfusion has implemented technology policies to protect personal information from misuse and loss

Table 15: Summary of the Assessment of Regulations Applied in PHR Systems.

**PHR**	**Standards/Regulations**
**Microsoft Health Vault [28]**	They comply with the HONcode principles for trustworthy health information. Microsoft HealthVault is not a covered entity as defined by HIPAA. They offer HealthVault solution providers which are HIPAA covered entities the opportunity to sign their HealthVault business associate agreement
**HealthButler [29]**	HONcode
**Google Health [30]**	Google Health is not covered by HIPAA, but the partners that are regulated by this are not required to comply with Google's Privacy Policy
**ZebraHealth [31]**	The system is in compliance with HIPAA

**NoMoreClipBoard.com [32]**	NoMoreClipboard.com was designed to support the privacy and security requirements of HIPAA while enabling the service to be used from any computer with Internet access
**My Doclopedia PHR [33]**	Doclopedia is not required to comply with the privacy rules implemented under HIPAA
**Healthy Circles [34]**	Not indicated
**Dr. I-Net [35]**	HONcode
**myHealthFolders [36]**	Not indicated
**Keas [37]**	Not indicated
**RememberItNow! [38]**	Not indicated
**MedsFile.com [39]**	Not indicated
**VIA [40]**	This system follows the principles and guidelines of HIPAA
**PatiensLikeMe [41]**	HONcode
**Telemedical.com [42]**	This Website subscribes to the HONcode principles
**My HealtheVet [43]**	The system is in compliance with HIPAA
**MedicAlert [44]**	Not indicated
**dLife [45]**	HONcode
**Juniper Health [46]**	Not indicated
**MyChart [47]**	PHI is individually identifiable under HIPAA if it includes the name, address, zip code, geographical codes, date of birth, other elements of dates, telephone or fax numbers, email address, social security number, insurance information, medical record number, member or account number, certificate/license numbers, voice or finger prints
**MediCompass [48]**	This PHR system meets and exceeds current industry standards for privacy, security and confidentiality including HIPAA. It subscribes to HONcode principles
**EMRy STICK [49]**	Not indicated
**myMediConnect; Passport MD [50]**	Although myMediConnect is not required to comply with HIPAA, they use HIPAA as a guideline for their policies and procedures
**iHealthRecord [51]**	Not indicated
